# Methodology and results of real-world cost-effectiveness of carfilzomib in combination with lenalidomide and dexamethasone in relapsed multiple myeloma using registry data

**DOI:** 10.1007/s10198-019-01122-6

**Published:** 2019-10-31

**Authors:** M. Campioni, I. Agirrezabal, R. Hajek, J. Minarik, L. Pour, I. Spicka, S. Gonzalez-McQuire, P. Jandova, V. Maisnar

**Affiliations:** 1 Economic Modeling Center of Excellence, Global Health Economics, Amgen (Europe) GmbH, Zug, Switzerland; 2 Department of Haematooncology, University Hospital Ostrava and Faculty of Medicine, University of Ostrava, 17. listopadu 1790, 708 52 Ostrava, Czech Republic; 3 Department of Hemato-Oncology, University Hospital Olomouc and Faculty of Medicine and Dentistry, Palacky University Olomouc, I. P. Pavlova 185/6, 779 00 Olomouc, Czech Republic; 4 Department of Internal Medicine, Hematology and Oncology, University Hospital Brno and Faculty of Medicine Masaryk Universit, Jihlavská 340/20, 625 00 Brno, Czech Republic; 5 Department of Internal Medicine, Charles University in Prague, First Faculty of Medicine and General Teaching Hospital, Katerinska 32, 121 08 Prague, Czech Republic; 6 Global Health Economics, Amgen (Europe) GmbH, Zug, Switzerland; 7 Amgen s.r.o, Prague, Czech Republic

**Keywords:** Carfilzomib, Multiple myeloma, Cost-effectiveness, Real world, ASPIRE, Registry of Monoclonal Gammopathies, I19

## Abstract

**Objective:**

To predict the real-world (RW) cost-effectiveness of carfilzomib in combination with lenalidomide and dexamethasone (KRd) versus lenalidomide and dexamethasone (Rd) in relapsed multiple myeloma (MM) patients after one to three prior therapies.

**Methods:**

A partitioned survival model that included three health states (progression-free, progressed disease and death) was built. Progression-free survival (PFS), overall survival (OS) and time to discontinuation (TTD) data for the Rd arm were derived using the Registry of Monoclonal Gammopathies in the Czech Republic; the relative treatment effects of KRd versus Rd were estimated from the phase 3, randomised, ASPIRE trial, and were used to predict PFS, OS and TTD for KRd. The model was developed from the payer perspective and included drug costs, administration costs, monitoring costs, palliative care costs and adverse-event related costs collected from Czech sources.

**Results:**

The base case incremental cost effectiveness ratio for KRd compared with Rd was €73,156 per quality-adjusted life year (QALY) gained. Patients on KRd incurred costs of €117,534 over their lifetime compared with €53,165 for patients on Rd. The QALYs gained were 2.63 and 1.75 for patients on KRd and Rd, respectively.

**Conclusions:**

Combining the strengths of randomised controlled trials and observational databases in cost-effectiveness models can generate policy-relevant results to allow well-informed decision-making. The current model showed that KRd is likely to be cost-effective versus Rd in the RW and, therefore, the reimbursement of KRd represents an efficient allocation of resources within the healthcare system.

**Electronic supplementary material:**

The online version of this article (10.1007/s10198-019-01122-6) contains supplementary material, which is available to authorized users.

## Introduction

Multiple myeloma (MM), generally considered incurable, is the second most common haematological malignancy and accounts for approximately 0.8% of all new cancer cases worldwide [1–3]. The incidence and survival of cancer patients, in general, as well as of MM in particular, have increased in the past few decades, and a similar trend has been observed for the economic burden of cancer management [4–7]. For this reason, and particularly under a situation of budget constraints that many healthcare decision-makers are facing, the value of cancer drugs is increasingly being scrutinised [7, 8].

Cost-effectiveness studies, along with other health economic studies such as budget impact analyses, represent essential tools that allow healthcare managers to make evidence-based decisions regarding the value and affordability of health technologies. Randomised controlled trials (RCTs) are the gold standard to identify relative treatment effects and are well suited to produce evidence for regulatory approval; [6], however, Sullivan et al. and Neyt et al. argue that results from cost-effectiveness analyses based solely on RCTs may not predict the benefits and costs of new treatments in real world (RW) patients and that these analyses should be supplemented with information collected from observational databases when available [6, 9]. In fact, there are differences between RCTs and the RW that may limit the applicability of economic models based on RCTs only in RW populations: potential differences in patient selection criteria (i.e. stricter inclusion and exclusion criteria in RCTs, in general, as compared with RW studies), treatment patterns and dosing, use of supportive care and extent of follow-up (i.e. patients’ adherence to treatment tends to be better in RCTs, as compared with RW studies), or differences in care across countries, particularly in the context of oncology, are some examples [6, 8, 10]. Observational databases, however, capture characteristics and outcomes of patients receiving treatment in real life: the Registry of Monoclonal Gammopathies (RMG), for instance, captures a wide range of data of MM patients in the Czech Republic, and comparisons across published studies demonstrate that differences exist between RCTs and the RW, e.g. outcomes of patients treated with lenalidomide and dexamethasone (Rd) are considerably lower in RW patients compared with those in recent RCTs [11–16]. Additionally, the limited time duration of RCTs pose an extra hurdle for the generalisation of economic model results in the RW, as the time horizon of economic models often requires extrapolation of clinical data well beyond the trial duration; [17] in registries and observational databases patients may be followed for longer periods and consequently the uncertainty around long-term estimates may be considerably lower than that obtained as a result of extrapolation of trial data [9, 17, 18]. Mullins et al. claim that this RW evidence is critical for coverage decisions by payers and treatment decisions by physicians and patients, and for that reason economic models that combine the strengths of both RCTs (i.e. relative treatment effects) and RW data [i.e. baseline risks such as progression-free survival (PFS) and overall survival (OS) in patients receiving the comparator treatment] may provide more relevant and less uncertain estimates than those based on RCTs only, as long as the evidence available from observational databases is robust and representative of the RW patient population [8, 9, 19, 20]. Therefore, this modelling approach is deemed to be appropriate to support well-informed decision-making in the RW, as it may minimise the risk of inefficient allocation of resources, including the chances of neglecting the access to more efficacious therapies erroneously considered not cost-effective, as well as the likelihood of inaccurate budget impact predictions [8, 9, 19, 20].

Several studies have reported the RW cost-effectiveness of cancer drugs combining data from RCTs and observational databases, reinforcing the validity of the approach described above. For instance, Seferina et al. estimated the RW cost-effectiveness of trastuzumab plus chemotherapy versus chemotherapy alone in early breast cancer combining RW outcomes for the trastuzumab arm with treatment effect estimates [expressed as hazard ratios (HRs) of trastuzumab versus control arm] from the HERA trial [21, 22]. Similarly, van Gils et al. analysed the RW cost-effectiveness of oxaliplatin in colon cancer, for which they combined published efficacy data from the MOSAIC trial with RW data from a Dutch population-based observational study [10]. Other studies have adopted a similar approach for the estimation of RW cost-effectiveness of health technologies, including disease areas other than cancer such as cardiovascular disease or chronic obstructive pulmonary disorder [23–26].

The aim of the present study was to estimate the RW cost-effectiveness of carfilzomib in combination with lenalidomide and dexamethasone (KRd) compared with Rd for the treatment of relapsed MM after one to three prior therapies. For this purpose observational data for Rd from the RMG in the Czech Republic were combined with treatment effect estimates from the ASPIRE trial, a randomised, open-label, multicentre, phase 3 study that evaluated the safety and efficacy of KRd compared with Rd in relapsed MM patients who had received one to three prior treatments [12, 15, 16].

## Methods

### Data sources

Real-world data for the Rd arm were collected from the RMG [16]. This database was set up in 2007 and captures all newly diagnosed MM patients treated in 19 Czech hospitals (16 hospitals reported relevant data at the time of data collection), covering approximately 80% of all newly diagnosed MM and monoclonal gammopathy of unknown significance (MGUS) patients in the Czech Republic [16]. The RMG is considered the most comprehensive database in Central Europe, and includes information on MM disease status and history (e.g. laboratory tests performed and results, disease stage), treatment received (e.g. specific regimen, time to treatment discontinuation [TTD], line of therapy) and outcomes (e.g. PFS and OS). Data from the intention-to-treat population in the ASPIRE RCT were also used to inform the cost-effectiveness model [15]. These two data sources were combined in such a way that baseline risks of events with Rd treatment were estimated from the RMG, whereas the relative treatment effects of KRd versus Rd were estimated from ASPIRE, as suggested and presented in the literature [9, 10, 21–26]. A comparison of baseline characteristics of Rd patients in RMG and ASPIRE are presented in the online resources (see Supplementary Table 1). Patients of the RMG registry were older, had worse performance status, were more likely to be refractory to prior bortezomib and immunomodulatory treatment, and were less likely to have received stem cell transplantation. Cost data were collected from Czech sources.

### Model structure

**Fig. 1:**
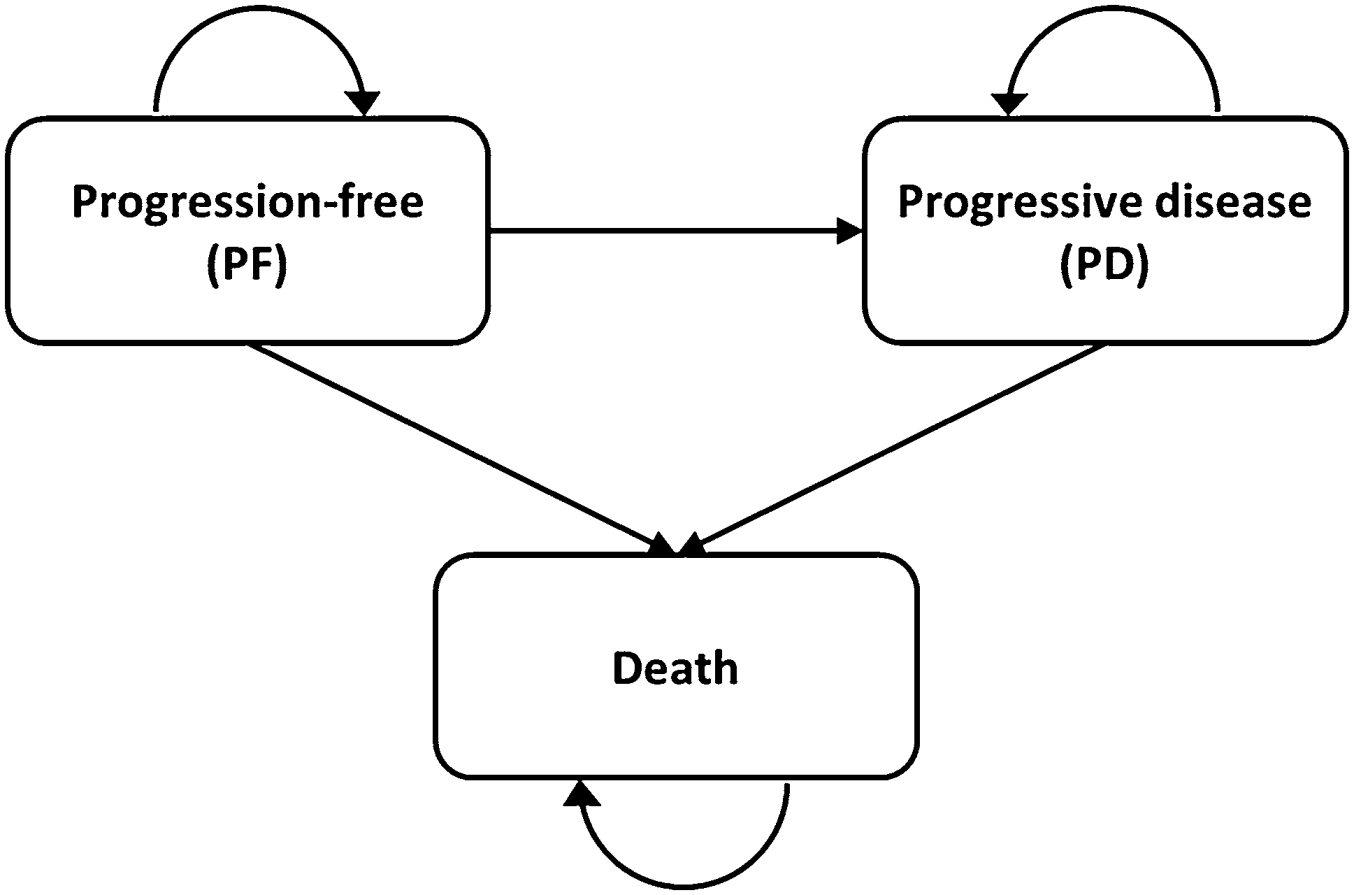
Model structure

A partitioned survival model was built with three mutually exclusive health states, i.e., progression-free (PF), progressive disease (PD) and death (Fig. 1). Transitions to the death state could occur from either the PF or PD health states, death being an absorbing state. The proportions of patients in each health state over time were estimated using the PFS and OS curves in each treatment arm. A cycle length of 28 days was implemented in line with the carfilzomib administration schedule [15]. This modelling approach has been extensively used for economic models in MM, including the cost-effectiveness model of KRd versus Rd from a US perspective authored by Jakubowiak et al. [27–33].

### Regimens

Rd was chosen as the only comparator treatment because it is the most widely used treatment regimen in relapsed MM after one to three prior therapies in the Czech Republic. Although bortezomib-based and pomalidomide treatments are also available, treatment patterns data demonstrate that the market share of Rd was approximately 70–75% in 2018 [34]. This comparator choice was supported by representatives of a local expert society (Czech Myeloma Group).

Carfilzomib was implemented in the model as per the cycle dosing in the carfilzomib summary of product characteristics (SPC) and ASPIRE study [15, 35]. Dosing for carfilzomib is based on body surface area (BSA), and the reference value considered for this analysis was 1.73 m^2^, in line with previous decisions by the State Institute for Drug Control in the Czech Republic (SÚKL) [36]. Dosing for Rd was based on the lenalidomide SPC [37]. Additional details are available elsewhere [15, 32].

### Treatment effectiveness

The PFS, OS and TTD curves for patients receiving Rd were estimated from the RMG. The RMG provided separate PFS, OS and TTD data on patients treated with Rd in second, third and fourth lines (2L, 3L and 4L), and median values are shown in Table 1. Kaplan–Meier (KM) curves for PFS and OS are provided in the online resources (Supplementary Figure 1 and 2).

**Table 1:** Median PFS, OS and TTD values for patients receiving Rd in the RMG

Outcome	2L (*n* = 113)	3L (*n* = 96)	4L (*n* = 15)
Median PFS, months (95% CI)	8.7 (7.3–10.1)	6.6 (5.3–8.0)	5.7 (1.6–9.7)
Median OS, months (95% CI)	26.2 (21.7–30.8)	12.6 (11.4–13.7)	10.6 (5.7–15.6)
Median TTD, months (95% CI)	7.2 (NA)	5.2 (NA)	3.8 (NA)

*1L* first line, *2L* second line, *3L* third line, *CI* confidence interval, *n* number of patients, *NA* not available, *OS* overall survival, *PFS* progression-free survival, *Rd* lenalidomide/dexamethasone, *RMG* Registry of Monoclonal Gammopathies, *TTD* time to discontinuation

Survival analyses were conducted according to the National Institute of Health and Care Excellence (NICE) guideline and parametric models were fitted to PFS, OS and TTD data in each line (exponential, Weibull, Gompertz, loglogistic, lognormal and generalised gamma models) [20]. The suitability of each model was assessed through visual comparison of the fit versus the corresponding KM curve, goodness-of-fit statistics (Akaike information criterion [AIC]), and plausibility of long-term extrapolations. The best fitting parametric models in each line were selected, and a weighted average of these curves was computed using the number of patients in each line in RMG (see Table 1) to derive PFS, OS and TTD baseline curves for the overall patients population with one to three prior lines.

The relative treatment effect estimates for KRd versus Rd expressed as HRs (for PFS and OS), were derived using ASPIRE patient-level data. PFS data were available from the first ASPIRE data cutoff in June 2014 (median follow-up 31 months), while mature OS data were available from a later data cut in April 2017 (median follow-up 67 months) [15, 38]. To assess the proportionality of the hazards, log-cumulative hazard plots were evaluated, along with tests of interaction between treatment effect and time with a Cox model [20, 32, 39]. The PFS and OS HRs of KRd versus Rd were calculated with separate multiple Cox models using a number of baseline characteristics as covariates to reduce potential imbalances between treatment arms [40]. Specifically, all covariates that were prespecified for subgroup analyses in ASPIRE were included in the initial models. Covariates to be included in the final models were identified by first testing each variable independently; it was assessed whether the variable was associated with the outcome (at a significance level of 0.2). Variables identified in this process were then trimmed one at a time (significance level of 0.1 or higher) with a stepwise variable selection procedure to derive the final PFS and OS model. This stepwise procedure examined the association between baseline covariates and outcomes (PFS and OS) as well as the effects of interaction between treatment and covariates by including treatment, each of the covariates and treatment-covariate interaction terms as predictor variables. The resulting PFS and OS HRs were applied to baseline risks derived from the RMG to estimate the PFS and OS curves for KRd, respectively (see online resources, Supplementary Table 2 and 3). The TTD curve for KRd was calculated applying the PFS HR to the Rd TTD curves from the RMG in order to simulate that the efficacy associated with a particular treatment may be associated with the amount of treatment received by patients.

### Health-state utilities

The RMG does not record preference-based utility data for MM patients, and these were not collected in the ASPIRE trial. For these reasons, utility inputs were estimated by combining utilities from the literature and trial-based patient-reported outcomes. The methodology for estimating these utilities and the utility values used in the model have been described by Jakubowiak et al. [32]. The impact of adverse events (AEs) on health-related quality of life was also considered as part of this evaluation by incorporating utility decrements (or disutilities) associated with each relevant AE taking into account the duration of the AE [29]. The approach adopted and the disutility values implemented have also been detailed by Jakubowiak et al. [32]. The implicit assumption associated with this approach was that utilities in RMG patients were considered to be similar to those in ASPIRE patients.

### Costs

The model was developed from the payer perspective, and costs from Czech sources were used to illustrate the current RW cost-effectiveness model. Costs were obtained in Czech korunas, and then translated into euros using the average exchange rate between June 11th, 2017, and December 11th, 2017 (1 EUR = 25.931 CZK) [41]. In line with the published literature, the following cost inputs were considered: drug costs, administration costs, monitoring costs, palliative care costs and AE-related costs [32, 33].

#### Initial drug costs

Drug prices were collected from the Czech list of reimbursed medicinal products as of December 1st, 2017 [42]. To calculate drug costs, mean weight or BSA of patients, available strengths (for a vial, capsule or tablet), price of a pack and the number of vials, capsules or tablets in a pack were considered. Also, in order to appropriately model the treatment acquisition costs based on the actual doses captured in the RMG registry, and in alignment with the literature, relative dose intensity (RDI) was applied to reflect the impact of dose reductions and interruptions on drug acquisition costs [43–47]. In the Rd arm, the RDI values were calculated from the RMG dividing the mean dose of lenalidomide per administration (in mg) by 25 mg (i.e. the maximum dose as per the lenalidomide label). For the KRd arm, RDI values from ASPIRE were used, as it represented the best source of evidence for patients receiving all three drugs in combination. Carfilzomib wastage was assumed to be negligible, due to the current availability of 60, 30 and 10 mg vials, and therefore the cost per mg was used in the model. Information on the dosing for each treatment, along with the RDI and the cost per cycle for each drug, are presented in Table 2.

**Table 2:** KRd and Rd drug costs

Treatment regimen	Regimen components	Unit	Unit cost (€)	Dosing schedule	RDI (%)	Drug cost per 28-day model cycle (€)
KRd	Carfilzomib (Cycle 1)	1 × 60 mg vial	1400.03	20 mg/m^2^ on Days 1 and 2, 27 mg/m^2^ on Days 8, 9, 15, and 16	91.0	5437
	Carfilzomib (Cycles 2-12)	1 × 60 mg vial	1400.03	27 mg/m^2^ on Days 1, 2, 8, 9, 15, and 16	91.0	5951
	Carfilzomib (Cycles 13 and beyond)	1 × 60 mg vial	1400.03	27 mg/m^2^ on Days 1, 2, 15, and 16	91.0	3967
	Lenalidomide	21 ×× 25 mg tablets	5116.65	25 mg orally on days 1-21	80.5	4119
	Dexamethasone	20 × 20 mg tablets	25.33	40 mg orally on days 1, 8, 15 and 22	82.8	8
Rd	Lenalidomide	21 × 25 mg tablets	5116.65	25 mg orally on days 1-21	88.2	4512
	Dexamethasone	20 × 20 mg tablets	25.33	40 mg orally on days 1, 8, 15 and 22	88.2	9

*KRd* carfilzomib/lenalidomide/dexamethasone, *Rd* lenalidomide/dexamethasone, *RDI* relative dose intensity

#### Subsequent treatment costs

Drug prices were collected from the Czech list of reimbursed medicinal products as of December 1st, 2017 [42]. The model considered that patients in the PD state may receive subsequent active treatments. Prior to receiving subsequent treatments, patients experience a treatment-free interval of three cycles (the same in both treatment arms) during which no treatment costs were applied [32]. The proportions of patients progressing and receiving subsequent treatments were estimated from the RMG: 54.1% of patients went on to receive subsequent treatments (the same in both treatment arms). These patients entered a ‘tunnel state’ consisting of a mix of treatments derived from patients captured in the RMG, whom were treated following the Czech Myeloma Group guidelines for MM (Table 3) [48]. The detailed proportions of patients receiving each subsequent treatment were collected from the RMG and are provided in the online resources (Supplementary Table 4). The RDI was assumed to be 100% for all subsequent treatments due to the lack of data, and overall duration for subsequent treatments was assumed to be 5 cycles for both KRd and Rd, based on data from the RMG (additional details are provided in the online resources; Supplementary Table 5).

**Table 3:** Unit costs and dosing schedule of subsequent treatments

Treatment	Unit	Unit cost (€)	Dosing schedule	Drug cost per 28-day model cycle (€)
Bortezomib (Actavis)^a^	1 × 3.5 mg vial	161.45	4 subcutaneous administrations; each administration of 1.3 mg/m^2^	415
Thalidomide^b^	30 × 50 mg tablets	80.98	28 oral administrations; each administration 100 mg	148
	30 × 100 mg tablets	158.11		
Cyclophosphamide (Endoxan)^c^	10 × 200 mg	26.46	28 intravenous administrations; each administration of 100 mg	37
	1 × 500 mg	6.62		
	1 × 1 g	13.23		
Pomalidomide (Imnovid)^d^	21 × 1 mg	8910.32	21 oral administrations; each administration of 4 mg	9329
	21 × 2 mg	9049.81		
	21 × 3 mg	9189.17		
	21 × 4 mg	9328.66		

^a^Bortezomib SPC provides information for a 3-week (21-day) long cycle, and frequency was transformed to a 4-week (28-day) long cycle

^b^Dosing schedule informed by expert opinion. Minimum cost per mg was chosen

^c^Dosing schedule informed by expert opinion. Alternatively, patients could also receive 300 mg/m^2^ on Day 1 and Day 15 of a 28-day cycle. Minimum cost per mg was chosen

^d^Minimum cost per mg was chosen

#### Administration costs

Carfilzomib and cyclophosphamide were assumed to be administered intravenously at the hospital (outpatient) at a cost of €27.19 per administration [49, 50]. Costs of oral and subcutaneous administrations were assumed to be zero and therefore no other drug was considered to incur any administration costs.

#### Monitoring costs

Monitoring items were derived from the NICE technology appraisal of panobinostat for MM, and included skeletal survey by X-ray, laboratory analyses (serum protein assessment, haematology, blood chemistry and thyroid function tests) and specialist visits [51]. Resource use was estimated from a study that involved seven centres of excellence for MM treatment in the Czech Republic, and costs were collected from the latest available health checklist published by the Ministry of Health [49]. These inputs yielded a figure of €31.46 for monitoring costs per patient per cycle, which was assumed to be the same in both treatment arms. Additional details are provided in the online resources (Supplementary Table 6).

#### Palliative care costs

All progressed patients that were not in either the treatment-free interval or receiving subsequent treatments were assumed to incur a standard cost for palliative care, with a cost per cycle of €1093 [52].

#### Adverse event costs

Adverse events were included in the model if they were Grade 3 or Grade 4 with an incidence equal or greater than 2% in ASPIRE. Monthly probabilities of AEs were calculated from the percentages of patients experiencing an AE over the course of the ASPIRE trial and from the meantime on treatment in ASPIRE (KRd = 88.1 weeks; Rd = 70.7 weeks). Patients were assumed to be at a constant risk of having an AE while on treatment in the PF state. Unit costs for AEs were identified from the list of Diagnosis Related Group (DRG) codes valid for 2017 [53]. Table 4 displays the monthly probabilities and unit costs of AEs included in the model.

**Table 4:** Estimated monthly probabilities of Grade 3 or Grade 4 adverse events and the unit costs (with the corresponding DRG code) of each adverse event

Adverse event	% Grade 3		% Grade 4		Unit cost (€)	DRG inpatient code
	KRd (%)	Rd (%)	KRd (%)	Rd (%)		
Blood and Lymphatic System Disorders						
Neutropenia	1.18	1.12	0.28	0.42	964.02	16341-3
Anaemia	0.34	0.51	0.09	0.08	1001.35	16331-3
Thrombocytopenia	0.37	0.42	0.37	0.21	964.02	16341-3
Gastrointestinal Disorders						
Diarrhoea	0.08	0.13	0.00	0.00	533.34	06371-3
Vomiting	0.00	0.00	0.00	0.00	716.59	17332
Respiratory, Thoracic and Mediastinal Disorders						
Dyspnoea	0.08	0.02	0.00	0.00	776.06	0411-3
General Disorders and Administration Site Conditions						
Fatigue	0.30	0.29	0.00	0.00	716.59	17332
Nervous System Disorders						
Peripheral neuropathy	0.08	0.10	0.00	0.00	716.59	17332

*DRG* Diagnosis Related Group, *KRd* carfilzomib/lenalidomide/dexamethasone, *Rd* lenalidomide/dexamethasone

### Discount rate

A discount rate of 3.0% per annum was applied for costs and outcomes, in line with the SÚKL methodological guidance [54].

### Time horizon

The median age at baseline in the RMG registry and ASPIRE study was 67 and 64 years, respectively, but patients as young as 49 and 31 years were included in the RMG registry and ASPIRE study, respectively [12, 15]. Therefore, a lifetime time horizon (40 years) was considered appropriate in the base case given the patients’ heterogeneity in terms of age at diagnosis. This time horizon would allow capturing all costs and consequences of all patients over their lifetime.

### Sensitivity analyses

Univariate deterministic sensitivity analyses (DSA) were conducted to test the effects of parameter uncertainty within the model. The model parameters were varied using 95% confidence intervals (CIs), if available; if these were not available, standard probability distributions were assigned to model parameters and lower and upper limits were calculated as the 2.5th and 97.5th percentile, respectively, assuming a standard error (SE) equal to 10% of the base case values. Lower and upper bounds of curve fit parameters were estimated with their corresponding variance–covariance matrices within a multinormal distribution. Probabilistic sensitivity analyses (PSA) were also conducted. Standard probability distributions were assigned to model parameters and 5000 Monte Carlo simulations were computed. Finally, a number of scenario analyses were performed: (1) only add-on therapy costs, i.e. carfilzomib costs, were considered, given that Rd has previously been appraised and recommended as a cost-effective treatment option, including in the Czech Republic; [55–57] (2) unadjusted PFS and OS HRs (i.e. the HR from the primary ASPIRE publication for PFS and the unadjusted HR estimated for OS using the data made available in April 2017); [15, 37] (3) same utilities for KRd and Rd arms, assuming KRd utilities for both arms; (4) time horizon of 20 years; (5) discount rate of 0% for both costs and outcomes, as per the SÚKL guidelines; [54] and (6) discount rate of 5% for both costs and outcomes, as per the SÚKL guidelines [54].

## Results

### Base case analysis

The survival analyses for PFS of patients receiving Rd in the RMG yielded the lowest AIC for the log-logistic curves in second- and third-line patients, and for the exponential curve in fourth-line patients. For OS, the exponential curve resulted in the lowest AIC in second- and fourth-lines, and for the log-logistic curve in third-line patients. For TTD, the Weibull curve was associated with the lowest AIC in second- and third-lines; the AIC of the Weibull curve in fourth-line patients was very similar to that of the lowest AIC (Gompertz), and for that reason the Weibull function was selected for estimating TTD in all three lines. AIC values for PFS, OS and TTD are reported in the online resources (Supplementary Table 7).

**Fig. 2:**
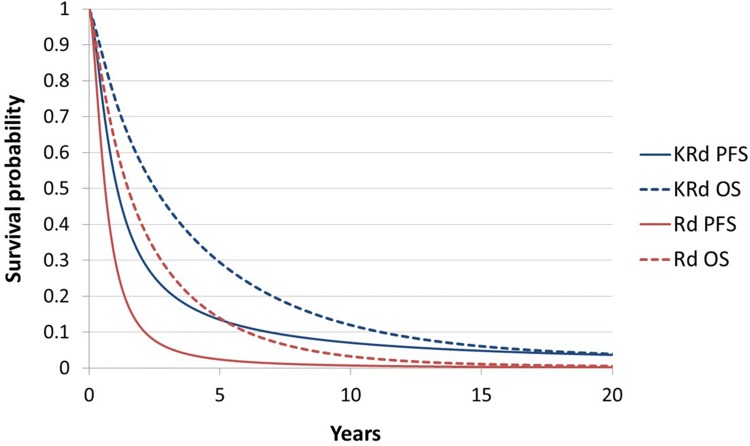
Progression-free survival and overall survival curves for Rd and KRd in the base case analysis

The results from the test of interaction between treatment effect and time (*p* = 0.08 for PFS; *p* = 0.41 for OS), and visual examination of the log-cumulative hazard plots suggested that the proportional hazards assumption was valid, as reported by Jakubowiak et al. [32]. The stepwise Cox models showed that there was no evidence of treatment-covariate interaction which, along with the lack of evidence of differences in relative treatment effects across subgroups reported by Stewart et al., supported the assumption that relative treatment effects observed in ASPIRE could be transferable to the RW setting [15, 32]. The stepwise Cox models identified a number of baseline covariates with a potential prognostic effect for predicting PFS and OS. For PFS, the following covariates were identified: baseline haemoglobin (higher risk of progression if < 105 g/L), baseline platelet count (higher risk if < 150 × 10^9^/L), baseline calcium level (higher risk if > 11.5 mg/dL), International Staging System (ISS) stage at diagnosis (higher risk for stage II compared with stage I and missing categories; similar risk for stage II and III patients), β-2 microglobulin level at stratification (higher risk if ≥ 2.5 mg/L), risk group as determined by fluorescent in situ hybridisation (higher risk for high risk patients compared with standard and unknown categories), prior bortezomib exposure (higher risk for patients with prior bortezomib exposure) and refractory to immunomodulatory agents in any prior regimen (higher risk for refractory patients). For OS, the following covariates were identified: sex (higher risk of death for male patients), baseline Eastern Cooperative Oncology Group (ECOG; higher risk for patients with ECOG of 2 compared with patients with ECOG of 1; similar risk for patients with values of 0 and 1), baseline haemoglobin (higher risk if < 105 g/L), baseline platelet count (higher risk if < 150 × 10^9^/L), baseline creatinine clearance (continuous variable), disease stage at diagnosis (higher risk for stage II compared with stage I), β-2 microglobulin level at stratification (higher risk if ≥ 2.5 mg/L) and refractory to immunomodulatory agents in any prior regimen (higher risk for refractory patients). The multiple Cox models showed statistically significant treatment effects for both PFS and OS: the PFS HR was equal to 0.641 (95% CI 0.526–0.781; *p* value < 0.001) and OS HR equal to 0.731 (95% CI 0.612–0.872; *p* value < 0.001). Given that the assumption of proportional hazards was considered appropriate, the HRs calculated from these analyses were applied to the PFS, OS (Fig. 2) and TTD curves of Rd to derive the corresponding KRd curves.

The base case ICER for KRd compared with Rd was €73,156 per QALY gained (Table 5). Patients on KRd incurred costs of €117,534 over their lifetime compared with €53,165 for patients on Rd. The QALYs gained were 2.63 and 1.75 for patients on KRd and Rd, respectively; the life years (Lys) gained were 3.42 and 2.43 for patients on KRd and Rd, respectively.

**Table 5:** Base case results

	Total costs (€)	Total LYs	Total QALYs	Incremental costs (€)	Incremental LYs	Incremental QALYs	ICER (€/QALY)
Rd	53,165	2.43	1.75	64,368	0.99	0.88	73,156
KRd	117,534	3.42	2.63				

*KRd* carfilzomib/lenalidomide/dexamethasone, *ICER* incremental cost-effectiveness ratio, *LY* life year, *QALYs* quality-adjusted life year, *Rd* lenalidomide/dexamethasone

Table 6 shows that the largest proportion of incremental costs is due to the increased treatment costs in the KRd arm. Higher costs of lenalidomide and dexamethasone in the KRd arm are a consequence of extending Rd treatment duration in the KRd arm compared with the Rd arm, due to a better response to treatment in KRd patients that allows patients to remain on therapy for longer. Costs of AEs and monitoring costs are also higher in the KRd arm due to patients staying longer in the PF state, as compared with patients receiving Rd treatment.

**Table 6:** Summary of predicted costs by item

Item	Cost KRd (€)	Cost Rd (€)	Increment (€)
Drug cost: carfilzomib	56,152	0	56,152
Drug cost: lenalidomide	41,273	36,069	5204
Drug cost: dexamethasone	84	71	13
Administration cost: carfilzomib	1414	0	1414
Adverse events costs	270	224	46
Monitoring costs	839	451	388
Subsequent treatments	1013	1216	− 203
Administration cost: subsequent treatments	107	128	− 21
Palliative care costs	16,382	15,006	1375
Total	117,534	53,165	64,368

*KRd* carfilzomib/lenalidomide/dexamethasone, *Rd* lenalidomide/dexamethasone

### Sensitivity analyses

**Fig. 3:**
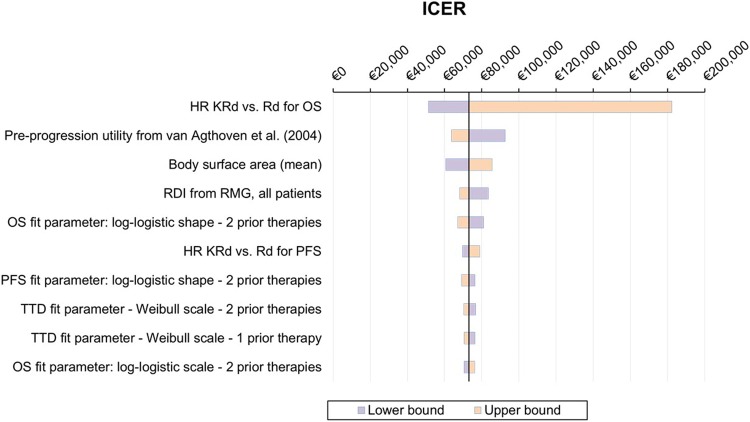
Tornado diagram illustrating the results of the univariate deterministic sensitivity analyses

Results of univariate DSA are presented in a form of a tornado diagram (Fig. 3). The ICER was most influenced by the OS HR, followed by the pre-progression utilities, BSA, RDI and the shape parameter of the log-logistic curve for OS in second-line patients. The model results were less sensitive to the TTD estimates and PFS HR.

**Fig. 4:**
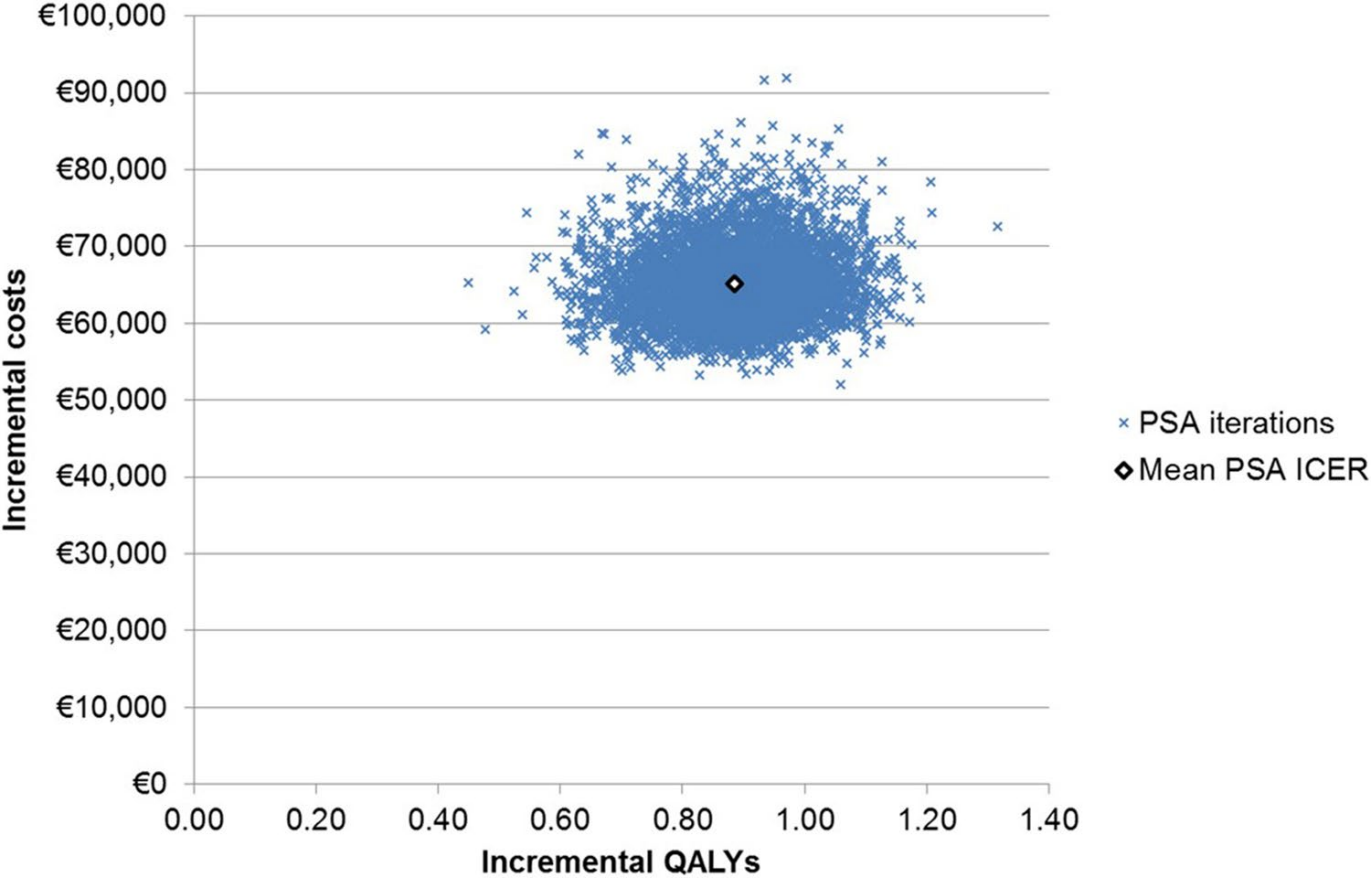
Incremental cost-effectiveness plane for KRd versus Rd

**Fig. 5:**
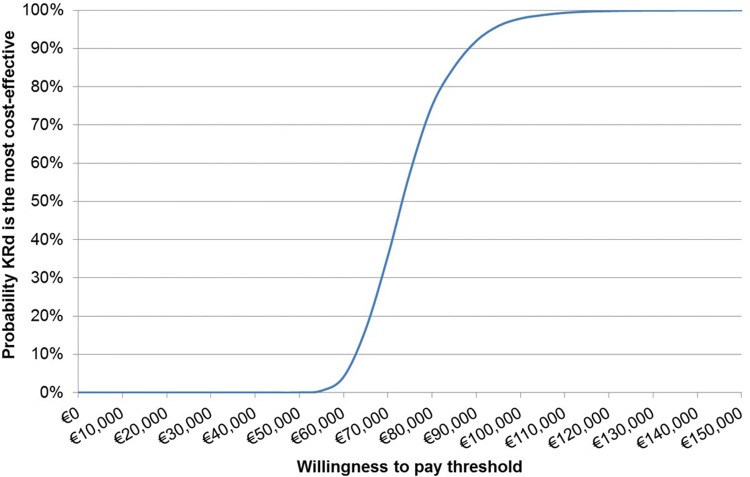
Incremental cost-effectiveness acceptability curve for KRd versus Rd

The results of the PSA are shown in Fig. 4. The scatter plot of incremental costs and QALYs shows that all simulations resulted in KRd being more effective and more costly than Rd, yielding an ICER very close to the base case ICER (€73,649 per QALY). The cost-effectiveness acceptability curve (Fig. 5) demonstrates that the probability of KRd being the most-effective intervention was highest at a willingness to pay threshold between €70,000 and €75,000 per QALY and above.

Results from scenario analyses are summarised in Table 7. Consideration of carfilzomib costs only resulted in a reduction of the ICER from €73,156 to €67,347 per QALY, while the implementation of the unadjusted PFS and OS HRs pushed the ICER up to €93,094 per QALY. Implementing discount rates of 0% for costs and outcomes reduced the ICER (€56,930 per QALY) compared with the base case, whereas assuming the same utilities for KRd and Rd arms, setting the time horizon at 20 years and assuming discount rates of 5% increased the ICER (€77,258, €80,703 and €83,807 per QALY, respectively).

**Table 7:** Results from scenario analyses

Scenario	KRd			Rd			Incremental costs (€)	Incremental LYs	Incremental QALYs	ICER (€/QALY)
	Total costs (€)	Total LYs	Total QALYs	Total costs (€)	Total LY	Total QALYs				
Base case	117,534	3.42	2.63	53,165	2.43	1.75	64,368	0.99	0.88	73,156
Carfilzomib costs only^a^	75,648	3.42	2.63	16,391	2.43	1.75	59,257	0.99	0.88	67,347
Unadjusted PFS and OS HRs^b^	112,651	3.12	2.39	53,165	2.43	1.75	59,485	0.69	0.64	93,094
Same utilities for KRd and Rd arms^c^	117,534	3.42	2.63	53,165	2.43	1.80	64,368	0.99	0.83	77,258
Time horizon of 20 years	117,410	3.31	2.53	52,954	2.41	1.73	64,456	0.90	0.80	80,703
Discount rate of 0% for both costs and outcomes	121,767	3.98	3.07	55,609	2.66	1.91	66,158	1.32	1.16	56,930
Discount rate of 5% for both costs and outcomes	115,126	3.16	2.42	51,871	2.31	1.67	63,255	0.84	0.75	83,807

*HR* hazard ratio, *ICER* incremental cost-effectiveness ratio, *KRd* carfilzomib/lenalidomide/dexamethasone, *LY* life year, *OS* overall survival, *PFS* progression-free survival; QALY, quality-adjusted life year; Rd, lenalidomide/dexamethasone

^a^Rd drug costs were excluded from both KRd and Rd arms

^b^The PFS HR was obtained from the primary ASPIRE publication. The OS HR from the April 2017 data cut was 0.794 (95% CI 0.667–0.945; *p* value < 0.001)

^c^Utilities of KRd assumed for both KRd and Rd arms

## Discussion

The current analysis evaluated the RW cost-utility of KRd versus Rd in relapsed MM patients that have received one to three prior therapies, resulting in an ICER of €73,156 per QALY gained in the base case. The cost-utility model developed for the analysis used a partitioned survival modeling approach which is employed in a significant proportion of economic evaluations of cancer therapies. Scientifically reputable health technology assessment (HTA) agencies such as NICE have repeatedly reviewed and confirmed the appropriateness of such model structure [29, 30]. The analysis was conducted from the payer perspective, and the Czech Republic was chosen to illustrate the model given the rich observational data sources available in the country.

For estimating the RW cost-effectiveness of KRd versus Rd, the baseline hazard of patients treated with Rd (PFS, OS and TTD) were calculated from the RMG, one of the most comprehensive and relevant registries capturing outcomes of MM patients [16]. The KRd versus Rd HRs from ASPIRE were applied to the baseline hazard to estimate the hazard of patients receiving KRd in the RW, assuming that the relative treatment effects observed in ASPIRE are applicable in the RW. Results from the phase 3 ASPIRE trial demonstrated that the relative treatment effects are consistent across a wide variety of subgroups of relapsed MM patients, and additional statistical analyses showed no significant treatment-covariate interaction in the ASPIRE patient population [15, 32]. This is regarded as a strong evidence base to support the applicability of trial HRs in the RW [9]. This methodology has been previously adopted for the estimation of RW cost-effectiveness of health technologies in oncology as well as other disease areas, such as cardiovascular and respiratory diseases; [10, 21, 22, 24–26] the approach has also been accepted by NICE, issuing a positive recommendation for evolocumab for treating primary hypercholesterolaemia or mixed dyslipidaemia in specific patient groups based on an economic model that combined baseline risks of cardiovascular disease from the Clinical Practice Research Datalink registry with reductions in cardiovascular events from a meta-analysis of RCTs [58].

Neyt et al. argue that combining observational data with evidence from RCTs is a solution for handling potential differences between RW patients and RCT patients: RCTs are the gold standard for estimating relative treatment effects, whereas observational databases capture baseline risks of patients treated in RW conditions, and therefore an analysis that combines the strengths of both observational and RCT data may result in results that are more relevant for policy purposes, compared with results obtained from data collected under ideal circumstances (i.e. RCTs) only. With regard to the current decision problem, the outcomes observed in ASPIRE were substantially better than those observed in the RMG: in ASPIRE, the median PFS and OS were 17.6 and 40.4 months, respectively, for patients receiving Rd; [15, 38] patients in the RMG, however, had median PFS and OS values of approximately 7.6 and 19.3 months, respectively (weighted values from Table 1). Similar differences were identified for treatment duration: the median TTD was 13.1 months in the Rd arm in ASPIRE, in contrast with the 6.1 months in the RMG (Table 1) [15, 38]. These dissimilarities between ASPIRE and the RMG are likely to arise from differences in patient characteristics, treatment selection and treatment patterns between the trial and the RW. For these reasons, and given the available evidence base, the use of registry data to inform baseline risks in economic models is considered to present healthcare managers with the most relevant information package for an appropriate decision-making and avoid unrealistic budget impact predictions caused by overestimating key variables such as treatment duration. This is particularly important in MM where a number of trials that enrolled patients across the world have consistently shown better outcomes and longer treatment duration than what is achieved in the RW [11–15].

The sensitivity analyses showed that the model is particularly sensitive to the parameters predicting and assumptions made around the relative treatment effect for OS associated with KRd versus Rd. However, considering that RW outcomes are not yet available for KRd, the base case is considered to represent a set of plausible assumptions.

In the current model, patients in the KRd arm were estimated to spend longer time in PFS compared with patients in the Rd arm, which in turn extended the use of lenalidomide and dexamethasone in the KRd arm (the cost of lenalidomide and dexamethasone was €41,273 versus €36,069 in the KRd and Rd arms, respectively; Table 6). Innovative therapies like carfilzomib tend to extend the use of costly therapies that have been considered cost-effective in the past (e.g. lenalidomide given on top of carfilzomib in the KRd regimen), and this could generate the perception that the innovative therapies are more expensive than they actually are [32, 56, 57]. The currently accepted methodology for cost-effectiveness analysis does not consider the new paradigm of oncology regimens administered in combination, which represents a major hurdle to demonstrate cost-effectiveness of innovative therapies. HTA agencies such as NICE have recognised these challenges and acknowledged that some innovative therapies may not even be cost-effective at zero price, but no practical solution has been proposed and widely accepted thus far [59]. For these reasons, one scenario analysis evaluated the cost-effectiveness of carfilzomib excluding the costs of lenalidomide and dexamethasone in both KRd and Rd arms, i.e. focusing the analysis on the introduction of carfilzomib only. The ICER was lower than that of the base case (€67,347 and €73,156 per QALY in the scenario analysis and base case, respectively), which is in line with the results shown by Jakubowiak et al. [32]. This approach was accepted by NICE in the technology appraisal of cinacalcet, where the costs of dialysis were excluded from the base case analysis [60, 61].

In RCTs, it is expected that the randomisation process will produce treatment groups that are balanced across the covariate levels. In reality, however, it is common to observe post hoc imbalances in covariates across treatment groups, which may have a confounding effect. In order to remove the between-patient variability associated with covariates not included as randomisation factors and increase the generalisability of the analyses, as well as allowing for the unbiased transferability to RW data, PFS and OS HRs estimated from ASPIRE were adjusted for a number of baseline covariates [32]. A scenario analysis was conducted to quantify the impact of covariate adjustment on cost-effectiveness results by implementing the unadjusted HRs from ASPIRE, and the ICER increased from €73,156 to €93,094 per QALY [15]. Nevertheless, the stepwise Cox models conducted on the ASPIRE patient-level data indicated that a number of covariates may have a prognostic effect on PFS and OS, and therefore the base case ICER is considered to be more precise and relevant for decision-making purposes.

Additional scenario analyses demonstrated the robustness of the model results. The assumption of equal utilities in the KRd and Rd arms, which represents a conservative assumption as described by Jakubowiak et al., only increased the ICER to €77,258 per QALY, and a similar effect on the ICER was observed when shortening the time horizon to 20 years (€80,703 per QALY) or setting the discount rate of both costs and outcomes at 5% (ICER of €83,807 per QALY). On the other hand, assuming a discount rate of 0% improved the cost-effectiveness of KRd considerably, yielding an ICER of €56,930 per QALY.

The analysis had various limitations associated with the underlying data and methods. Firstly, the review of the literature to identify some input parameters for the cost-effectiveness model was not systematic. All inputs were, however, obtained from relevant data sources (either from the pivotal clinical trial ASPIRE or local data sources in the Czech Republic) and therefore it is considered that the impact of not having conducted a systematic literature review for all input parameters is minimal. This strategy is aligned with other RW CE studies in the literature [10, 21, 22, 62]. The PFS, OS and TTD curves were derived from data collected during a period in which, in the Czech Republic, patients were treated with lenalidomide only up to a maximum cumulative dose of 4200 mg [56]. The model, however, assumed that patients would be treated with lenalidomide until progression, in line with the most recent decision in October 2016 by SÚKL on lenalidomide reimbursement, and costs of lenalidomide and dexamethasone were implemented accordingly [57]. The outcomes that would have been observed if lenalidomide and dexamethasone had been given until progression may have been better than those captured in the RMG and used in the current model, and therefore the outcomes generated in the current model may be an underestimation. On the other hand, no hard stop at eight cycles (i.e. equivalent to a cumulative dose of 4200 mg assuming no dose reductions and no missed doses) or any time point afterwards was observed in the TTD curves from the RMG, indicating that the impact of the 4200 mg cap may not be sizable. With regard to AE rates, the model included rates estimated from the ASPIRE frequencies of AEs. No data on AEs were available from the RMG and therefore no further adjustment was conducted. This represents a further limitation, although the impact of AE costs on the cost-effectiveness of KRd is minimal (i.e. the incremental cost of AEs is only 0.07% of the total incremental costs of KRd compared with Rd; see Table 6). The last PFS and OS events in patients captured in the RMG happened at nearly 5 years; the KM estimates showed a probability of remaining progression-free of approximately 5% and a probability of survival of approximately 20% at about 5 years (see online resources; Supplementary Figure 1 and 2). The long-term extrapolation of PFS and OS may be seen as a key contributor to the model uncertainty particularly considering the extent of the time horizon in the base case but, taking into account the maturity of the RMG data, this long-term extrapolation is not deemed to have a large impact on results. Besides, in a recent retrospective analysis of long-term PFS and OS data of Rd patients in the RMG registry, the median PFS and OS was estimated to be 9.0 months and 18.5 months, respectively[62]. PFS and OS at 6 years was < 5% and 20%, respectively. These values are very closely in line with the predictions of our model, therefore, we believe the PFS and OS predictions can be considered valid. Additionally, a scenario analysis looked into the impact of shortening the time horizon to 20 years and demonstrated that the choice of time horizon does not have a large impact on the cost-effectiveness results. Other limitations, such as the uncertainty around the utility estimates, have been discussed by Jakubowiak et al. [32].

The cost-effectiveness analysis by Jakubowiak et al. compared KRd versus Rd in relapsed MM from a US perspective, with an ICER of $107,520 per QALY [32]. The authors estimated that patients treated with KRd would benefit from 1.99 incremental LYs and 1.67 incremental QALYs compared with Rd, in contrast with the incremental 0.99 LYs and 0.88 QALYs estimated in the current model [32]. Larger differences can be observed when absolute LYs and QALY estimates are compared, despite the similar relative improvement in LYs and QALYs between the two analyses [32]. This reinforces the value of using RW data in cost-effectiveness analyses to avoid estimations that diverge from observed outcomes in the RW. However, these seemingly disparate results can be primarily explained by one key difference in the modelling approach between the two models: the data source used for calculating the PFS, OS and TTD curves. Jakubowiak et al. derived these curves for both KRd and Rd arms by fitting joint parametric models to the ASPIRE trial data; registry data (collected from the US Surveillance, Epidemiology, and End Results registry) were only used for the extrapolation of the Rd OS curve after the time of the last death event in the Rd arm in ASPIRE, and the OS HR was then used to estimate the corresponding OS curve for patients in the KRd arm.

In summary, this analysis showed that cost-effectiveness models of health technologies in the RW can generate policy-relevant results when the strengths of both RCTs and powerful observational databases are combined. The current model showed that KRd is likely to be cost-effective versus Rd in the RW population (MM patients with one to three prior therapies), with an ICER of €73,156 per QALY and these results, along with the cost-effectiveness analysis conducted by Jakubowiak et al., confirm that KRd is likely to be cost-effective versus Rd both in the clinical and RW settings [32]. Therefore, the reimbursement of KRd for this patient population represents an efficient allocation of resources within the healthcare system.

## Electronic supplementary material

Below is the link to the electronic supplementary material.

Supplementary material 1 (DOCX 155 kb)
