# Supplementary material for: Methodology and results of real-world cost-effectiveness of carfilzomib in combination with lenalidomide and dexamethasone in relapsed multiple myeloma using registry data
Source: Eur J Health Econ. 2019 Oct 31;21(2):219–33. doi: 10.1007/s10198-019-01122-6 (PMC7072050; doi:10.1007/s10198-019-01122-6)
Supplement: Supplementary file 1 — Supplementary material 1 (DOCX 155 kb) [file 10198_2019_1122_MOESM1_ESM.docx]

**Online Resources**

Supplementary Fig. 1 Progression-free survival curves of patients receiving Rd in the RMG

**
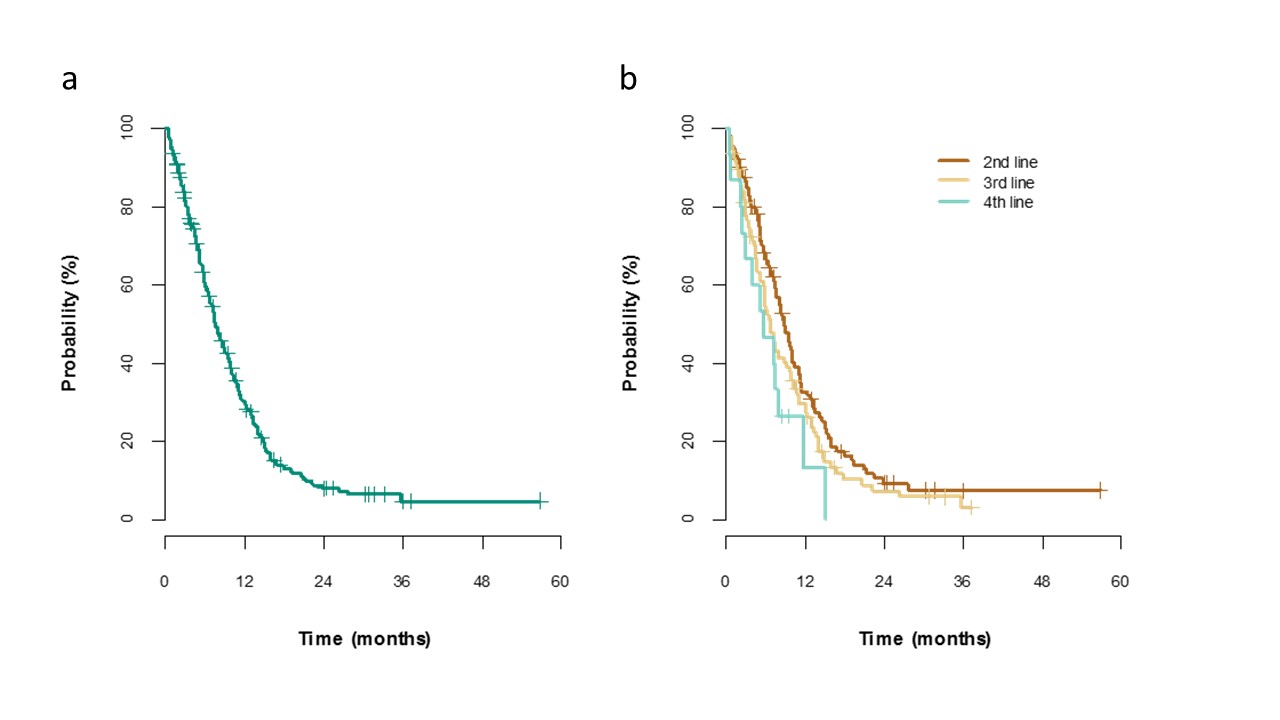
**

Rd, lenalidomide/dexamethasone; RMG, Registry of Monoclonal Gammopathies.

1. Progression-free survival of all patients with one to three prior lines.
2. Progression-free survival of patients, per treatment line.

Supplementary Fig. 2 Overall survival curves of patients receiving Rd in the RMG


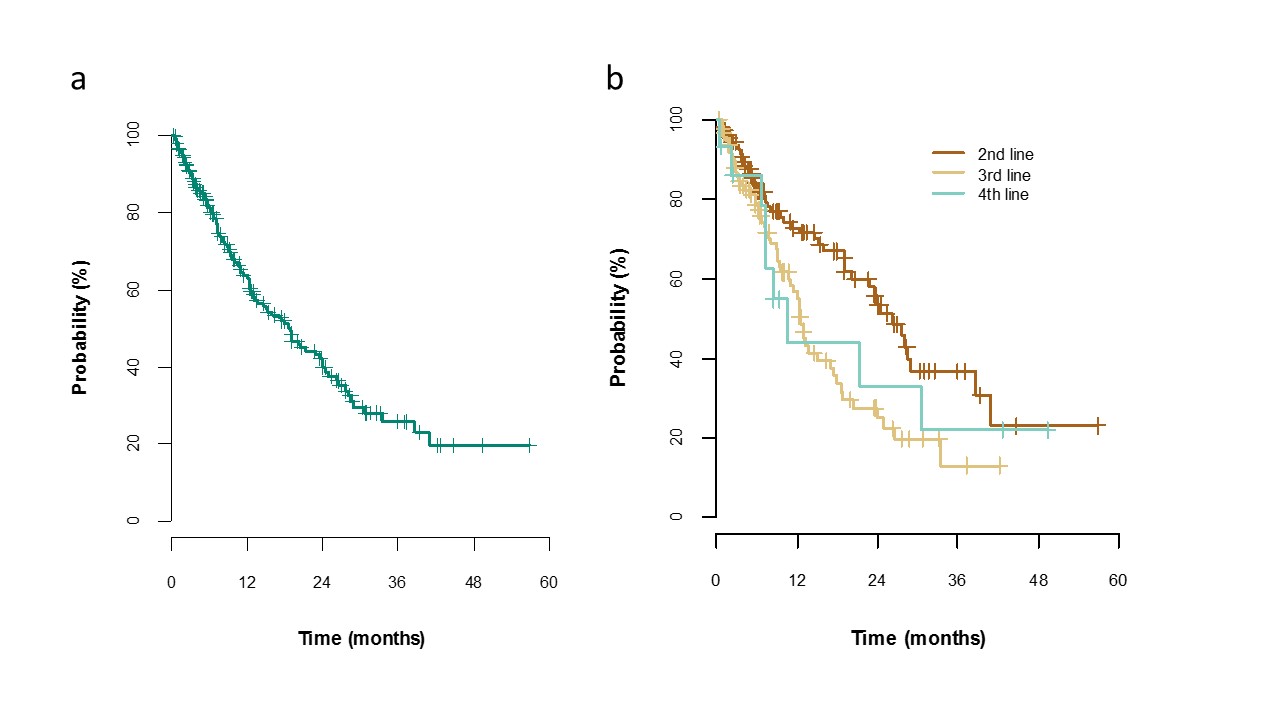


Rd, lenalidomide/dexamethasone; RMG, Registry of Monoclonal Gammopathies.

1. Overall survival of all patients with one to three prior lines.
2. Overall survival of patients, per treatment line.

Supplementary Table 1 Comparison of baseline characteristics in RMG and ASPIRE patients

| **Descriptive statistics** | **Second Line  RMG / ASPIRE**  **(n=113/n=157)** | **Third Line  RMG / ASPIRE**  **(n=96/n=139)** | **Fourth Line  RMG / ASPIRE**  **(n=15/n=100)** |
| --- | --- | --- | --- |
| **Age (median years)** | 69 / 66 | 66 / 64 | 76 / 65 |
| **Sex (n, % male)** | 47.8% / 51.6% | 52.1% / 69.1% | 40.0% / 55.0% |
| **Status Performance (ECOG/WHO)** |  |  |  |
| 0 | 8.2% / 38.9% | 6.5% / 51.1% | 6.7% / 43.0% |
| 1 | 60.9% / 53.5% | 70.7% / 40.3% | 53.3% / 46.0% |
| 2 | 21.8% / 7.6% | 18.5% / 8.6% | 40.0% / 11.0% |
| 3 | 7.3% / - | 4.3% / - | 0.0% / - |
| 4 | 1.8% / - | 0.0% / - | 0.0% / - |
| **Beta2 microglobulin (mg/l)** |  |  |  |
| <2.5 | 17.8% / 15.3% | 19.7% / 24.5% | 25.0% / 85.0% |
| ≥2.5 | 82.2% / 82.8% | 80.3% / 74.8% | 75.0% / 13.0% |
| **Creatinine level (median, umol/l)** | 87 / 75 | 93 / 86 | 121 / 74 |
| **Hemoglobin level (median, g/l)** | 115 / 109 | 111 / 116 | 117 / 107 |
| **ISS at diagnosis** |  |  |  |
| Stage 1 | 23.1% / 18.5% | 30.2% / 23.7% | 46.7% / 12.0% |
| Stage 2 | 38.9% / 23.6% | 40.7% / 20.1% | 6.7% / 29.0% |
| Stage 3 | 38.0% / 39.5% | 29.1% / 39.6% | 46.7% / 44.0% |
| **Prior Velcade exposure** | 61.9% / 46.5% | 94.8% / 75.4% | 100.0% / 82.0% |
| **Refractory to** ^1^: |  |  |  |
| IMiD | 17.6% / 12.1% | 34.3% / 24.5% | 58.3% / 35.0% |
| Velcade | 38.6% / 3.8% | 39.4% / 18.0% | 46.7% / 27.0% |
| **SCT status (yes)** ^2^ | 22.1% / 49.7% | 38.5% / 70.5% | 26.7% / 53.0% |

Note: Percentages may not add up to 100% due to rounding or missing data.

Supplementary Table 2 Multiple Cox regression analysis of PFS, detailed output

| **Covariate** | **Subgroup** | **Reference Group** | **Hazard Ratio** | | | **p-value**  **(2-sided)** |
| --- | --- | --- | --- | --- | --- | --- |
|  |  |  | **Point Estimate** | **95% CI** | |  |
| Treatment | KRd | Rd | 0.641 | 0.526 | 0.781 | <0.0001 |
| Baseline Hemoglobin Level Category | ≥ 105 g/L | < 105 g/L | 0.658 | 0.531 | 0.814 | 0.0001 |
| Baseline Platelet Level Category | ≥ 150 x10^9^/L | < 150 x10^9^/L | 0.665 | 0.535 | 0.825 | 0.0002 |
| Baseline Corrected Calcium Level Category | <= 11.5 mg/dL | >11.5 mg/dL | 0.610 | 0.347 | 1.073 | 0.0863 |
| Disease Stage at Initial Diagnosis | Missing | II | 0.771 | 0.553 | 1.073 | 0.1232 |
| Disease Stage at Initial Diagnosis | I | II | 0.730 | 0.533 | 1.001 | 0.0510 |
| Disease Stage at Initial Diagnosis | III | II | 0.986 | 0.777 | 1.251 | 0.9091 |
| β2 Microglobulin Level Category | < 2.5 mg/L | ≥ 2.5 mg/L | 0.790 | 0.603 | 1.034 | 0.0863 |
| Risk Group as Determined by FISH | Standard | High | 0.642 | 0.476 | 0.866 | 0.0037 |
| Risk Group as Determined by FISH | Unknown | High | 0.667 | 0.499 | 0.893 | 0.0065 |
| Prior Bortezomib Exposure (used for randomization stratification) | No | Yes | 0.745 | 0.603 | 0.919 | 0.0061 |
| Refractory to prior IMiD Regimen | No | Yes | 0.529 | 0.425 | 0.657 | <0.0001 |
| Abbreviations: CI = confidence interval; FISH = fluorescent in situ hybridization; IMiD = immunomodulatory drug; Source: Amgen, data on file. | | | | | | |

Supplementary Table 3 Multiple Cox regression analysis of OS, detailed output

| **Covariate** | **Subgroup** | **Reference Group** | **Hazard Ratio** | | | **p-value**  **(2-sided)** |
| --- | --- | --- | --- | --- | --- | --- |
|  |  |  | **Point Estimate** | **95% CI** | |  |
| Treatment | KRd | Rd | 0.731 | 0.612 | 0.872 | 0.001 |
| Sex | Male | Female | 1.185 | 0.989 | 1.419 | 0.066 |
| ECOG | 1 | 0 | 0.990 | 0.820 | 1.196 | 0.917 |
| ECOG | 2 | 0 | 2.035 | 1.525 | 2.716 | 0.000 |
| Baseline Hemoglobin Level Category | ≥ 105 g/L | < 105 g/L | 0.643 | 0.531 | 0.780 | 0.000 |
| Baseline Platelet Level Category | ≥ 150 x109/L | < 150 x109/L | 0.604 | 0.498 | 0.733 | 0.000 |
| Baseline Creatinine Clearance | Continuous variable | |  | 0.994 | 0.991 | 0.998 |
| Disease Stage at Initial Diagnosis | Missing | II | 1.232 | 0.931 | 1.632 | 0.145 |
| Disease Stage at Initial Diagnosis | I | II | 1.220 | 0.944 | 1.577 | 0.128 |
| Disease Stage at Initial Diagnosis | III | II | 1.155 | 0.839 | 1.591 | 0.377 |
| β2 Microglobulin Level Category | < 2.5 mg/L | ≥ 2.5 mg/L | 1.490 | 1.146 | 1.937 | 0.003 |
| Refractory to prior IMiD Regimen | Yes | No | 1.518 | 1.238 | 1.861 | 0.000 |
| Abbreviations: CI = confidence interval; ECOG = Eastern Cooperative Oncology Group; IMiD = immunomodulatory drug; Source: Amgen, data on file. | | | | | | |

Supplementary Table 4 Percentage of patients receiving each subsequent treatment after progression, per treatment line, in the RMG

|  | **After 2L (n=62)** | **After 3L (n=53)** | **After 4L (n=3)** |
| --- | --- | --- | --- |
| Bortezomib | 37.1% | 35.8% | 0.0% |
| Lenalidomide | 11.3% | 9.4% | 66.7% |
| Thalidomide | 17.7% | 26.4% | 0.0% |
| Cyclophosphamide plus dexamethasone | 19.4% | 9.4% | 0.0% |
| Pomalidomide plus dexamethasone | 0.0% | 7.5% | 0.0% |
| Bortezomib plus thalidomide | 0.0% | 0.0% | 0.0% |
| Other^a^ | 14.5% | 11.5% | 33.3% |
| 2L, second line; 3L, third line; 4L, fourth line; n, number of patients; RMG, Registry of Monoclonal Gammopathies. ^a^The cost of “other” treatments was assumed to be the minimum cost among all the subsequent treatments. | | | |

Supplementary Table 5 Duration of subsequent treatments after progression, per treatment line, in the RMG

|  | **After 2L (n=62)** | **After 3L (n=53)** | **After 4L (n=3)** |
| --- | --- | --- | --- |
| Duration (weeks) | 21.9 | 14.1 | 31.7 |
| 2L, second line; 3L, third line; 4L, fourth line; n, number of patients; RMG, Registry of Monoclonal Gammopathies. | | | |

Supplementary Table 6 Monitoring costs

| **Item** | **Proportion of patients tested** | **Test frequency (per cycle)** | **Unit cost (€)** | **Cost per 28-day cycle (€)** |
| --- | --- | --- | --- | --- |
| Skeletal survey by x-ray | 53.85% | 0.05 | 8.60 | 0.21 |
| Serum protein assessment | 100% | 1.11 | 2.43 | 2.71 |
| Lab results - haematology | 100% | 2.02 | 2.51 | 5.05 |
| Lab results - blood chemistry | 100% | 1.75 | 2.31 | 4.05 |
| Lab results - thyroid function test | 100% | 1.75 | 2.31 | 4.05 |
| Specialist visit (haematologist) | 100% | 2.26 | 6.83 | 15.39 |

Supplementary Table 7 AIC values associated with PFS, OS and TTD curves of patients receiving Rd in the RMG, per treatment line

|  | **PFS** | | | **OS** | | | **TTD** | | |
| --- | --- | --- | --- | --- | --- | --- | --- | --- | --- |
|  | **2L** | **3L** | **4L** | **2L** | **3L** | **4L** | **2L** | **3L** | **4L** |
| Exponential | 299.47 | 267.59 | 43.64 | 220.21 | 219.81 | 42.65 | 276.93 | 244.03 | 38.56 |
| Weibull | 297.23 | 267.15 | 44.61 | 221.77 | 219.75 | 44.36 | 231.29 | 221.72 | 34.45 |
| Gompertz | 301.46 | 269.58 | 44.07 | 221.91 | 221.51 | 43.55 | 235.42 | 230.83 | 34.25 |
| Log-logistic | 291.41 | 262.77 | 46.71 | 222.64 | 218.69 | 43.52 | 248.95 | 226.74 | 37.12 |
| Log-normal | 297.21 | 263.64 | 46.83 | 222.62 | 219.34 | 43.84 | 256.74 | 237.29 | 37.93 |
| Generalised gamma | 295.17 | 264.25 | NA^a^ | 223.58 | 220.26 | 45.77 | 232.12 | 223.22 | 35.96 |
| 2L, second line; 3L, third line; 4L, fourth line; NA, not available; OS, overall survival; PFS, progression-free survival; Rd, lenalidomide/dexamethasone; RMG, Registry of Monoclonal Gammopathies; TTD, time to discontinuation. ^a^Could not be computed. | | | | | | | | | |
